# Clinical comparative analysis of culture-proven bacterial keratitis according to prior topical steroid use: a retrospective study in a tertiary referral center of South Korea

**DOI:** 10.1038/s41598-023-41588-2

**Published:** 2023-09-02

**Authors:** Chan-Ho Cho, Nam Hyeon Choi, Sang-Bumm Lee

**Affiliations:** 1 Department of Ophthalmology, Haeundae Paik Hospital, Inje University College of Medicine, 875, Haeun-daero, Haeundae-gu, Busan, 48108 South Korea; 2 Nune Eye Hospital, 2179, Dalgubeol-daero, Jung-gu, Daegu, 41940 South Korea; 3 Department of Ophthalmology, Yeungnam University College of Medicine, 170, Hyunchung-ro, Nam-gu, Daegu, 42415 South Korea

**Keywords:** Outcomes research, Corneal diseases, Bacterial infection

## Abstract

This study analyzed the clinical characteristics of patients exposed to topical steroids before bacterial keratitis diagnosis (the prior topical steroid use, PS group), and compared these with those of the non-exposed group (the no prior topical steroid use, NPS group). We retrospectively analyzed 194 patients (PS, 34; NPS, 160) with culture-proven bacterial keratitis between 2007 and 2016. The microbiological profiles, epidemiology, predisposing factors, clinical characteristics, and treatment outcomes of PS and NPS were compared, and the risk factors for surgical intervention were evaluated. *Pseudomonas* spp. and *Staphylococcus* spp. were the most common isolates in PS and NPS, respectively, and no significant difference in the strain distribution between the two groups were observed. Significant differences were observed between PS and NPS for previous ocular surface disease (41.2%: 23.8%), initial BCVA < 0.1 (70.6%: 49.4%), epithelial defect size ≥ 5 mm^2^ (64.7%: 41.2%), epithelial healing time > 14 days (55.9%: 37.3%), and surgical intervention (23.5%: 8.8%). Prior topical steroid use, strong steroid use, and long-term steroid use groups were included in significant risk factors for surgical intervention. Previous exposure to topical steroids before the diagnosis of bacterial keratitis was associated with a worse initial clinical presentation and treatment outcomes. Additional multicenter studies should be conducted in the future.

## Introduction

Infectious keratitis is the leading cause of sight-threatening ocular disease, and is associated with various predisposing factors, including corneal trauma, previous ocular disease, and contact lenses^1^. Prior topical steroid use is an important risk factor for infectious keratitis. Although topical steroids are generally used for various ophthalmic diseases to control ocular inflammation^2^, steroid use promotes the proliferation of bacteria, fungi, and acanthamoeba, and can act as a potential factor in causing or exacerbating corneal infections^3–6^.

From a therapeutic point of view, four randomized controlled trials, including the Steroid Corneal Ulcer Trial (SCUT), have been reported as prospective studies on the therapeutic effect of topical steroids in bacterial keratitis^7–10^. These trials compared treatment results for topical steroids or placebo following the use of antibiotics for more than 24–48 h after a culture-proven diagnosis of bacterial keratitis. The results of the SCUT revealed that topical steroid use did not have a negative effect on treatment outcomes other than delayed epithelial regeneration, and no significant increase in adverse reactions such as surgical treatment and corneal perforation was observed^7^.

However, cases of infection exacerbation after topical corticosteroid use prior to the culture-proven diagnosis of infectious keratitis were not included in these prospective studies^7,9,10^. Meanwhile, in tertiary hospitals, referral cases due to corneal infection exacerbation after topical steroid use are common. Topical steroid use prior to an infectious keratitis diagnosis may cause temporary relief for corneal inflammation, thus concealing typical corneal presentation and making diagnosis and treatment difficult^4,11,12^. Therefore, retrospective evaluation of the clinical characteristics and treatment outcomes of infectious keratitis in patients previously exposed to topical steroid use is important.

In the case of fungal and acanthamoeba keratitis, topical steroid use before the diagnosis of infectious keratitis has been reported as a risk factor for poor treatment outcomes^5,6,13–19^. However, studies on the distribution of microorganisms, clinical characteristics, and treatment outcomes according to prior use of topical steroids with a focus on bacterial keratitis are limited^20^. Therefore, we conducted a retrospective comparative study of patients with culture-proven bacterial keratitis according to prior topical steroid use at a tertiary referral center in South Korea.

## Methods

### Study design

This was a retrospective, consecutive case series. The medical records of patients with culture-proven bacterial keratitis who were hospitalized and treated at the Yeungnam University Hospital between January 2007 and December 2016 were analyzed. Yeungnam University Hospital is a major tertiary referral hospital in Daegu Metropolitan City, South Korea, and covers the rural areas of Gyeongsangbuk-do Province.

The inclusion criteria for this study were a diagnosis of culture-positive bacterial keratitis and hospitalization for treatment. The exclusion criteria were a diagnosis of microbiologically-proven fungal or acanthamoeba keratitis, or bacteria and fungi were simultaneously isolated from the culture. Patients who used antifungal, antiviral, or anti-amoebic topical drugs were used for treatment were also excluded.

The overall cases were divided into the prior topical steroid use (PS) group, comprising those who had been using topical steroids for any reason at the time of initial presentation to our hospital and the no PS (NPS) group, comprising those with no prior topical steroid use. Microbiological profiles, baseline epidemiology, predisposing factors, clinical characteristics, and treatment outcomes were evaluated and compared between the PS and NPS groups.

For subgroup analysis, the PS group was divided into two categories based on: duration of prior steroid use and potency of steroid use. First, according to the duration of prior steroid use, the patients were divided into long- and short-term groups. The long-term group comprised cases of topical steroid use for 14 days or more prior to visiting our hospital, and the short-term group comprised cases of topical steroid use for less than 14 days prior to visiting our hospital. Second, based on the potency of the steroids, these were divided into strong and weak groups. The strong steroid group comprised patients who used loteprednol, prednisolone, or dexamethasone as a topical steroid prior to visiting our hospital, and the weak steroid group included those who use fluorometholone as a topical steroid prior to visiting our hospital^21^. The clinical features and treatment results were compared between the long- and short-term groups and the strong and weak steroids in terms of epidemiology, predisposing factors, initial clinical characteristics, and treatment outcomes.

### Ethical approval

This study complied with the principles outlined in the Declaration of Helsinki and was approved by the Institutional Review Board of Yeungnam University Hospital (No. 2020-11-038), Republic of Korea. The Institutional Review Board of our institution waived off the requirement of informed consent because obtaining consent from the patients during research was not possible and the risk to patients was very low as per the Bioethics and Safety Act of the Republic of Korea (Chapter 3, Article 16, Paragraph 3, Act No. 14839. Enforcement Date: July 26, 2017).

### Epidemiological investigation

Epidemiological characteristics such as sex, age, residential area, seasonal distribution, and symptom duration were investigated. Symptom duration was defined as the interval between symptom onset and the initial visit to our institution. Predisposing factors included previous ocular surface disease (OSD), previous ocular surgery, underlying systemic diseases, ocular trauma, contact lens use, and prior topical antibiotic use.

### Initial clinical characteristics

The location and size of corneal lesions, depth of infiltration, presence of hypopyon, and best-corrected visual acuity (BCVA) at the initial visit were investigated. The locations of the corneal lesions within half the radius of the center and limbus were classified as center and peripheral, respectively. Corneal lesion size was determined by measuring the corneal epithelial defect size (EDS) and was calculated as the area of a rectangle multiplied by the longest diameter of the epithelial defect measured using a slit lamp microscope ruler and the diameter perpendicular to it, as described by Mukerji et al.^22^ The depth of infiltration was categorized as either superficial (less than two-thirds of the depth of the stroma) or deep (more than two-thirds of the depth of the stroma). The BCVA at the initial visit was categorized under one of two groups, ≥ 0.1, or < 0.1, based on the Snellen visual acuity (VA). The mean VA was calculated by converting it to the logarithm of the minimum angle of resolution (logMAR), with hand motion, light perception, and no light perception defined as 2.6, 2.9, and 3.1, respectively^23^.

### Bacterial culture and identification

To identify the causative bacteria, specimens were collected through corneal scrapings from all patients before the administration of empirical antibiotics, and smear and culture tests were performed. Topical anesthesia was induced with 0.5% proparacaine hydrochloride (Alcaine^®^, Alcon, Fort Worth, TX, USA) before obtaining corneal scrapings. Corneal scrapings were obtained from all patients using a No.15 Bard-Parker knife (Bard-Parker Co., Danbury, CT, USA). Conjunctival swabs were collected simultaneously for all patients using sterile cotton-tipped swabs. The scrapings were smeared on glass slides and Gram staining was performed. To rule out fungal keratitis, KOH smear specimens were obtained from the margins and base of the ulcers, and placed within a marked area on a glass slide. One drop of 10% KOH was placed on the smear and a clean coverslip was added. Corneal scrapings were stained using Gram stain and acid-fast bacilli methods and inoculated on a variety of solid and liquid media that supported the growth of bacteria and fungi. Thioglycollate broth, blood agar, MacConkey agar, and Sabouraud’s dextrose agar media were used. The media were incubated for an appropriate period under the atmospheric conditions required for each medium and examined daily for the growth of the organisms. Cultured bacteria were identified using an automatic microbial analyzer (VITEK system; BioMérieux-Co, Lyon, France).

### Drug therapy

For empirical treatment, fortified eye drops of 2% tobramycin and cephalosporin antibiotics (5% cefamandole, 2007–2010; 5% ceftazidime, 2011–2016) were administered, commercially available fluoroquinolone antibiotic eye drops (0.5% moxifloxacin, Vigamox^®^, Alcon, Fort Worth, TX, USA) were administered every 1 h. Second-generation cephalosporins and aminoglycosides were administered systemically. If the clinical findings improved after follow-up, antibiotic use was continued, regardless of the susceptibility of the microorganisms. If the clinical course worsened, the antibiotics were changed according to the antibiotic susceptibility results. If topical steroid use was identified at the initial visit, the dose was gradually reduced and discontinued within a few days.

### Treatment outcomes

Treatment outcomes were assessed after 3 months or at treatment completion and were evaluated based on the epithelial healing time (EHT), duration of hospitalization, complications, surgical intervention, and final BCVA. The completion date of epithelial healing was defined as the period from the initial visit to the complete recovery of the epithelium. For statistical analysis, the final BCVA was classified as < 0.1 or ≥ 0.1, according to the Snellen chart.

### Statistical analysis

Data were analyzed using IBM SPSS Statistics for Windows (version 25.0; IBM Corp., Armonk, NY, USA). Chi-squared and Fisher’s exact tests were used to assess categorical data. Independent sample *t* tests were used to compare the mean values. Statistical significance was set at *p* < 0.05.

The risk of surgical intervention was evaluated using a *Z*-score obtained by performing a two-proportion *Z*-test on each independent variable. A risk factor analysis of the total cases was performed to determine which independent variable, including prior topical steroid use, had a significant effect on surgical intervention. The *Z*-score indicated the distance from the mean value of the normal population, and each independent variable is a multiple of the standard deviation. The *Z*-score indicated the relative position of statistical significance. When the conditions for each specified independent variable were satisfied, the *Z*-score was set as positive. The values corresponding to the 95%, 99%, and 99.9% confidence intervals of the Z-score were defined as ± 1.96, ± 2.58, and ± 3.29, respectively^24^.

## Results

### Enrolled patients

**Figure 1:**
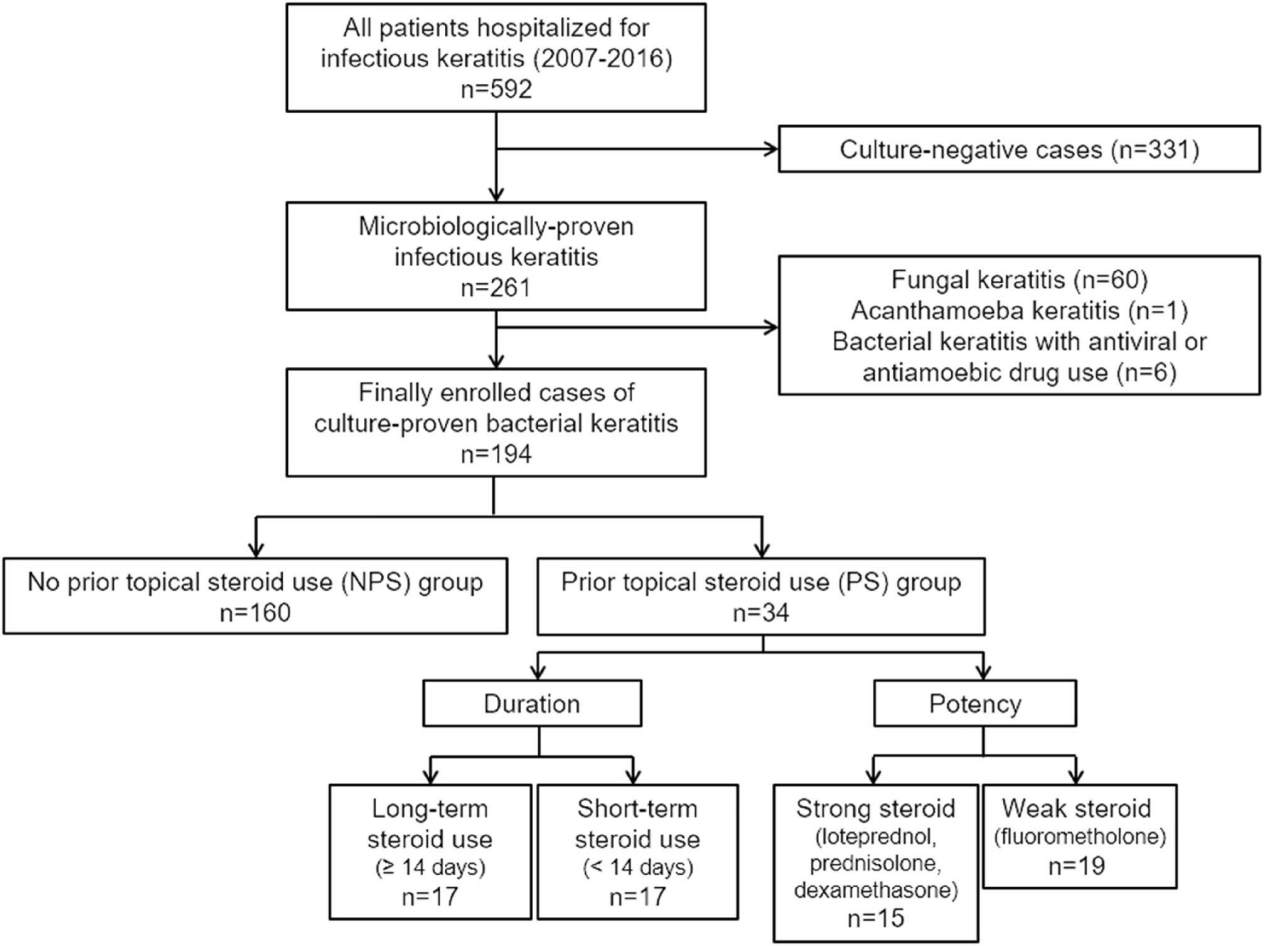
Flowchart of patient enrollment and group classification in the study.

A total of 592 patients with infectious keratitis were admitted to Yeungnam University Hospital during the study period. Overall, 331 patients who were culture-negative, 60 with culture-proven fungal keratitis, 1 with acanthamoeba keratitis, and 6 with bacterial keratitis treated with antiviral or anti-amoeba topical drug were excluded from the study. Finally, 194 patients with culture-positive bacterial keratitis were included in the study. Among them, 34 eyes (17.5%) were included in the PS group and 160 eyes (82.5%) were included in the NPS group. In the PS group, 17 eyes (50.0%) were assigned to the long- and short-term groups, respectively. Further, based on the potency of the prior topical steroid, 19 eyes (55.9%) and 15 eyes (44.1%) were assigned to the weak steroid and strong steroid groups, respectively (Fig. 1).

### Epidemiologic characteristics and predisposing factors

No significant differences in mean age, sex, age ≥ 60 years, residence, seasonal distribution, or symptom duration ≥ 7 days between the PS and NPS groups were observed (Table 1).

**Table 1:** Baseline epidemiology and predisposing factors of bacterial keratitis according to prior topical steroid use. Values are presented as number (%) or mean ± standard deviation.

Characteristics	PS (n = 34)	NPS (n = 160)	*p*-value (χ^2^ test)
Epidemiology			
Age, years	51.1 ± 21.8	53.8 ± 22.1	0.508^a^
Range (median, IQR)	6–78 (56.5, 35)	1–92 (58, 34)	
≥ 60	15 (44.1)	78 (48.8)	0.623
Male sex	17 (50.0)	81 (50.6)	0.947
Rural residency	16 (47.1)	72 (45.0)	0.827
Seasonal distribution			0.778
Spring (Mar–May)	11 (32.4)	49 (30.6)	
Summer (Jun–Aug)	8 (23.5)	39 (24.4)	
Autumn (Sep–Nov)	7 (20.6)	44 (27.5)	
Winter (Dec–Feb)	8 (23.5)	28 (17.5)	
Symptom duration ≥ 7 days	17 (50.0)	60 (37.5)	0.176
Predisposing factors			
Corneal trauma	19 (55.9)	94 (58.8)	0.758
Contact-lens wear	3 (8.8)	36 (22.5)	0.071
Previous ocular surface disease	14 (41.2)	38 (23.8)	0.037
Herpetic keratitis	4 (11.8)	9 (5.6)	0.249^b^
Exposure keratitis	2 (5.9)	6 (3.8)	0.631^b^
Previous corneal ulcer	4 (11.8)	9 (5.6)	0.249^b^
Bullous keratopathy	2 (5.9)	5 (3.1)	0.355^b^
Others^c^	3 (8.8)	11 (6.9)	0.715^b^
Previous ocular surgery	5 (14.7)	31 (19.4)	0.525
Diabetes mellitus	3 (8.8)	17 (10.6)	1.000^b^
Prior topical antibiotics use	18 (52.9)	73 (45.6)	0.438

IQR, interquartile range; NPS, no prior topical steroid use; PS, prior topical steroid use.

^a^*p*-values were calculated using independent t-tests.

^b^*p*-values were calculated using Fisher’s exact test.

^c^Includes dry eye disease with superficial punctate keratopathy (PS, one case; NPS, four cases), band keratopathy (NPS, two cases), acute keratoconjunctivitis (PS, one case; NPS one case), atopic keratoconjunctivitis (PS, one case; NPS, one case), Stevens-Johnson syndrome (NPS, one case), Mooren’s ulcer (NPS, one case), and recurrent corneal erosion (NPS, one case).

Corneal trauma (PS, 55.9%; NPS, 58.8%) was the most common predisposing factor in both groups. The proportion of patients with previous OSD (41.2% vs. 23.8%, *p* = 0.037) was significantly higher in the PS group than in the NPS group. Contact lens use was less common in the PS group than in the NPS group (8.8% vs. 22.5%, *p* = 0.071). Further, no significant differences in corneal trauma, previous ocular surgery, diabetes, or prior topical antibiotic use between the two groups were observed (Table 1).

### Microbiological profiles

A total of 227 isolates were identified from 194 patients, including 40 isolates from the PS group and 187 isolates from the NPS group. A total of 65 (28.6%) gram-positive and 162 (71.4%) gram-negative bacteria were identified in the total group. Notably, *Staphylococcus* spp. and *Pseudomonas* spp. (47/227, 20.7%, respectively), followed by *Enterobacter* spp. (42/227, 18.5%) were the most common bacteria (Table 2).

**Table 2:** Microbiological test results according to prior topical steroid use.

Identified organisms	PS (n = 34)	NPS (n = 160)	*p*-value (χ^2^ test)
Gram-positive			
* Staphylococcus* species	5 (14.7)	42 (26.3)	0.154
* Enterococcus* species	2 (5.9)	8 (5.0)	0.688^a^
* Streptococcus* species	2 (5.9)	6 (3.8)	0.631^a^
Gram-negative			
* Pseudomonas* species	9 (26.5)	38 (23.8)	0.737
* Enterobacter* species	7 (20.6)	35 (21.9)	0.869
* Stenotrophomonas* species	2 (5.9)	25 (15.6)	0.177^a^
* Serratia* species	4 (11.8)	15 (9.4)	0.750^a^
* Acinetobacter* species	4 (11.8)	7 (4.4)	0.105^a^
* Achromobacter* species	0 (0.0)	5 (3.1)	0.589^a^
* Delftia* species	1 (2.9)	1 (0.6)	0.321^a^
* Klebsiella* species	1 (2.9)	1 (0.6)	0.321^a^
* Ochrobactrum* species	1 (2.9)	0 (0.0)	0.175^a^
* Proteus* species	1 (2.9)	0 (0.0)	0.175^a^
* Morganella* species	1 (2.9)	0 (0.0)	0.175^a^
* Citrobacter* species	0 (0.0)	1 (0.6)	1.000^a^
* Leclercia spp.*	0 (0.0)	1 (0.6)	1.000^a^
* Moraxella* species	0 (0.0)	1 (0.6)	1.000^a^
* Pantoea* species	0 (0.0)	1 (0.6)	1.000^a^
Total^b^	40 (100.0)	187 (100.0)	

NPS, no prior topical steroid use; PS, prior topical steroid use.

^a^*p*-values were calculated using Fisher’s exact test.

^b^Twenty-nine eyes had polymicrobial infection (PS, five eyes; NPS, twenty-four eyes); two isolates were observed in 26 eyes, three isolates were observed in two eyes, and four isolates were observed in one eye. *E. aerogenes, S. marcescens* (two cases); *E. cloacae, S. aureus* (two cases); *E. cloacae, S. maltophilia* (two cases); *P. aeruginosa, S. maltophilia* (two cases); *A. xylosoxidans, P. putida* (one case); *A. baumannii, S. epidermidis* (one case); *A. baumannii, S. maltophilia* (one case); *E. faecalis, E. cloacae* (one case); *E. faecalis, S. aureus* (one case); *E. faecium, P. putida* (one case); *M. catarrhalis, P. stutzeri* (one case); *P. aeruginosa, A. baumannii* (one case); *P. aeruginosa, E. aerogenes* (one case); *P. aeruginosa, S. epidermidis* (one case); *P. putida, A. denitrificans* (one case); *P. putida, S. aureus* (one case); *S. marcescens, E. cloacae* (one case); *S. marcescens, E. faecium* (one case); *S. marcescens, K. pneumoniae* (one case); *S. marcescens, S. maltophilia* (one case); *S. epidermidis, E. cloacae* (one case); *S. epidermidis, P. stutzeri* (one case); *E. cloacae, A. baumannii, E. aerogenes* (one case); *E. cloacae, E. aerogenes, S. epidermidis* (one case); *S. marcescens, A. xylosoxidans, S. epidermidis, E. cloacae* (one case).

In the PS group, *Pseudomonas* spp. (26.5%) was the most common, followed by *Enterobacter* spp. (20.6%), and *Staphylococcus* spp. (14.7%). In the NPS group, *Staphylococcus* spp. (26.3%) was the most common, followed by *Pseudomonas* spp. (23.8%), and *Enterobacter* spp. (21.9%). The proportion of gram-negative bacteria was higher than that of gram-positive bacteria in both the PS and NPS groups (73.5% vs. 65.0%, *p* = 0.339). Polymicrobial infection was observed in five eyes (14.7%) in the PS group and 24 eyes (15.0%) in the NPS group (*p* = 0.965) (Table 2).

### Initial clinical characteristics and treatment outcomes

A corneal EDS of ≥ 5 mm^2^ (64.7% vs. 41.7%, *p* = 0.013) was more common in the PS group than in the NPS group. No significant differences were observed between the two groups in terms of deep stromal infiltration (8.8% vs. 11.9%, *p* = 0.771) or hypopyon (26.5% vs. 24.4%, *p* = 0.797). The mean initial BCVA was significantly worse in the PS group than in the NPS group (1.77 ± 0.95 logMAR vs 1.32 ± 1.05 logMAR, *p* = 0.023), and the proportion of patients with an initial BCVA of < 0.1 (Snellen) was also significantly higher in the PS group than in the NPS group (70.6% vs. 49.4%, *p* = 0.024) (Table 3).

**Table 3:** Initial clinical characteristics and treatment outcomes of bacterial keratitis according to prior topical steroid use.

Characteristics	PS (n = 34)	NPS (n = 160)	*p*-value (χ^2^ test)
Initial clinical characteristics			
Central corneal lesion	24 (70.6)	86 (53.8)	0.072
Epithelial defect size ≥ 5 mm^2^	22 (64.7)	66 (41.2)	0.013
Deep infiltration	3 (8.8)	19 (11.9)	0.771^a^
Hypopyon	9 (26.5)	39 (24.4)	0.797
Initial BCVA, logMAR^c^	1.77 ± 0.95	1.32 ± 1.05	0.023^b^
< 0.1, Snellen^c^	24 (70.6)	78 (49.4)	0.024
Treatment outcomes			
Final BCVA, logMAR^d^	1.12 ± 1.10	0.76 ± 1.04	0.080^b^
< 0.1, Snellen^d^	12 (37.5)	36 (24.7)	0.138
Decreased BCVA^d^	3 (9.4)	12 (8.2)	0.735^a^
Epithelial healing time > 14 days^e^	19 (55.9)	57 (37.3)	0.045
Duration of hospitalization, days	11.7 ± 5.6	9.6 ± 4.9	0.025^b^
Surgical intervention	8 (23.5)	14 (8.8)	0.031^a^
AMT	6 (17.6)	10 (6.3)	0.040^a^
Tarsorrhaphy	0 (0.0)	1 (0.6)	1.000^a^
Scleral lamellar graft	0 (0.0)	1 (0.6)	1.000^a^
Conjunctival flap	1 (2.9)^f^	0 (0.0)	0.175^a^
Penetrating keratoplasty	1 (2.9)	0 (0.0)	0.175^a^
Evisceration	1 (2.9)	2 (1.3)	0.441^a^

AMT, amniotic membrane transplantation; BCVA, best-corrected visual acuity; logMAR, logarithm of the minimum angle of resolution; NPS, no prior topical steroid use; PS, prior topical steroid use.

^a^*p*-value calculated using Fisher’s exact test.

^b^*p*-value calculated using independent t-test.

^c^Total n = 192; two patients with NPS who could not check visual acuity were excluded.

^d^Total n = 178; 16 cases were excluded due to follow-up loss or inability to check visual acuity (PS, two cases; NPS, 14 cases).

^e^Total n = 187; seven patients with NPS were excluded due to loss to follow-up.

^f^In this patient, evisceration was performed because the course worsened after the conjunctival flap surgery.

The mean final BCVA improved compared with that at the first visit in both groups (both *p* < 0.001), and the proportion of patients with a BCVA of < 0.1 after treatment was also significantly reduced compared with that at the initial visit (PS: 70.6% → 37.5%, *p* = 0.007; NPS: 49.4% → 24.7%, *p* < 0.001). No significant differences in the mean final BCVA were observed between the two groups (1.12 logMAR vs 0.76 logMAR, *p* = 0.080). Compared to the initial BCVA, the final BCVA decreased in 3 eyes in the PS group and in 12 eyes in the NPS group (9.4% vs. 8.2%, *p* = 0.735) (Table 3).

The proportion of patients with EHT > 14 days was significantly higher in the PS group than in the NPS group (55.9% vs. 37.3%, *p* = 0.045). The mean hospitalization duration was significantly longer in the PS group than in the NPS group (11.7 days vs. 9.6 days, *p* = 0.025). Surgical treatment was performed in 8 (23.5%) and 14 eyes (8.8%) in the PS and NPS groups, respectively (*p* = 0.031). Amniotic membrane transplantation was the most frequently performed surgical treatment in both groups (17.6% vs. 6.3%, *p* = 0.040) (Table 3).

### Comparison according to duration and potency of prior topical steroid use within the PS group

**Figure 2:**
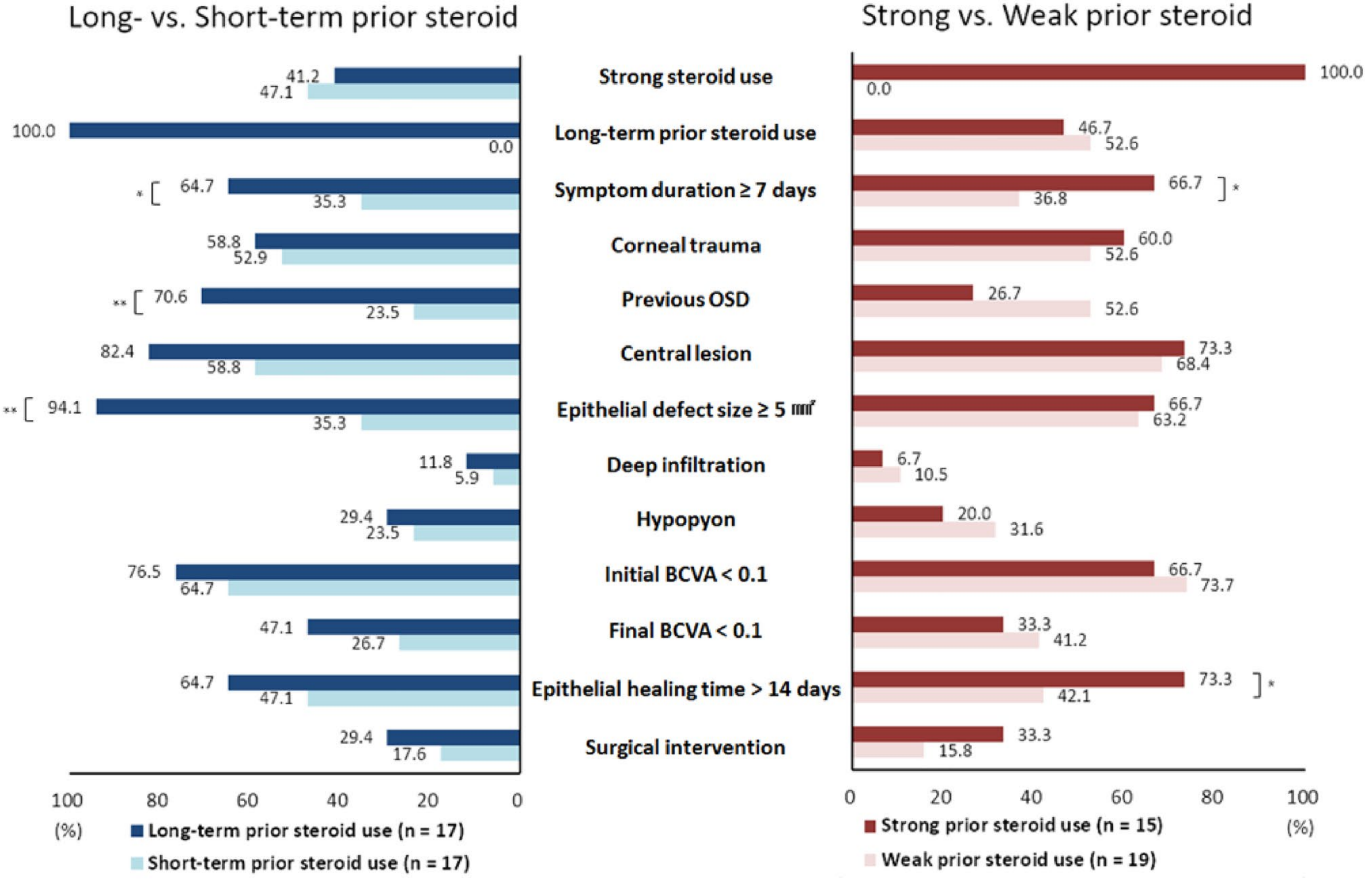
Comparison of clinical features according to the duration of prior topical steroid use and the potency of prior topical steroid use in the PS group of culture-proven bacterial keratitis. Long-term steroid = use of topical steroids for 14 days or more prior to visit; Short-term steroid = use of topical steroids for less than 14 days prior to visit; Strong steroid = use of loteprednol, prednisolone, or dexamethasone; Weak steroid = use of fluorometholone. Values are presented as proportion (%). BCVA, best-corrected visual acuity; OSD, ocular surface disease; PS, prior topical steroid use. ^*^p < 0.1, ^**^p < 0.05.

In the analysis based on the duration of steroid use, the long-term group had a higher proportion of patients with symptom duration ≥ 7 days (64.7% vs. 35.3%, *p* = 0.086), previous OSD (70.6% vs. 23.5%, *p* = 0.006), and EDS ≥ 5 mm^2^ (94.1% vs. 35.3%, *p* < 0.001) than the short-term group. No significant difference in strong steroid use (41.2% vs. 47.1%, *p* = 0.730), hypopyon (29.4% vs. 23.5%, *p* = 1.000, Fisher’s exact test), or surgical intervention (29.4% vs. 17.6%, *p* = 0.688) between the long- and short-term groups was observed (Fig. 2).

In the analysis based on steroid potency, the strong steroid use group had a relatively higher proportion of patients with symptom duration ≥ 7 days (66.7% vs. 36.8%, *p* = 0.084) and EHT ≥ 14 days (73.3% vs. 42.1%, *p* = 0.069) than the weak steroid use group, with no significant difference. No significant difference in the distribution of long-term prior steroid use (46.7% vs. 52.6%, *p* = 0.730), previous OSD (26.7% vs. 52.6%, *p* = 0.127), EDS ≥ 5 mm^2^ (66.7% vs. 63.2%, *p* = 0.832), deep infiltration (6.7% vs. 10.5%, *p* = 1.000, Fisher’s exact test), hypopyon (20.0% vs. 31.6%, *p* = 0.697), final BCVA < 0.1 (33.3% vs. 41.2%, *p* = 0.647), and surgical intervention (33.3% vs. 15.8%, *p* = 0.417) between the strong and weak steroid use groups was observed (Fig. 2).

### Risk factors leading to surgical intervention

**Figure 3:**
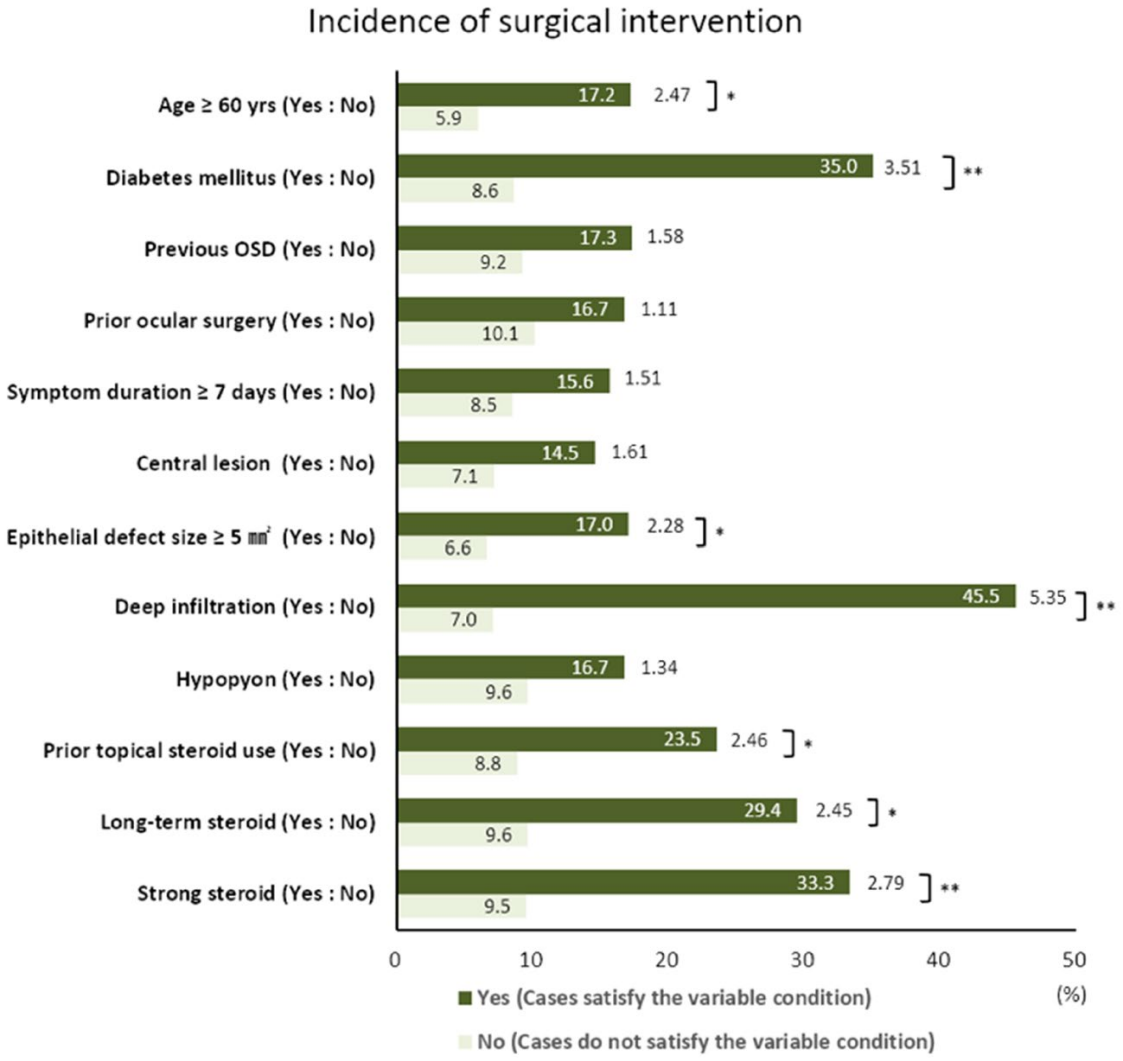
Risk factors leading to surgical intervention (n = 22) in the total group of patients with culture-proven bacterial keratitis (n = 194) analyzed using two-proportion *Z*-test. Values are presented as proportion (%, in the bar graph) or *Z*-score (outside the bar graph). OSD, ocular surface disease. ^*^p < 0.05 (*Z* > 1.96), ^**^p < 0.01 (Z > 2.58).

A two-proportion *Z*-test in the total group revealed that deep infiltration (*Z* = 5.35), diabetes (*Z* = 3.51), age ≥ 60 years (*Z* = 2.47), and EDS ≥ 5 mm^2^ (*Z* = 2.28) were significant risk factors for surgical intervention. Among prior topical steroid-related factors, prior topical steroid use (*Z* = 2.46), strong steroid use (Z = 2.79), and long-term steroid use (*Z* = 2.45) were significant risk factors for surgical intervention (Fig. 3).

## Discussion

The results of this study showed no significant differences in the distribution of causative isolates between the two groups. The PS group had a higher proportion of patients with previous OSD and poorer initial clinical characteristics, including a poorer initial BCVA and larger EDS, than the NPS group. The PS group experienced worse disease progression, including delayed epithelial healing and more surgical treatments, than the NPS group. In the PS group, the long-term group had a significantly higher proportion of patients with a previous OSD and larger epithelial defects than the short-term group, and the strong steroid group showed a relatively delayed hospital visit and epithelial healing time compared with the weak steroid group.

In this study, 17.5% of patients used topical steroids before their first visit to our hospital. In several reported studies, the proportion of prior steroid exposure in infectious keratitis has been reported to vary, with 3.1% of bacterial keratitis cases in Paris, France^25^; 7% of infectious keratitis cases in Japan^5^; and 41.3% of contact lens keratitis cases in Singapore^26^. In addition, a study of keratitis including acanthamoeba conducted in Nottingham, UK, reported that chronic steroid use before diagnosis was observed in 40% of cases^27^. Fluorometholone (55.9%) was the most commonly used topical steroid in the present study. In a Japanese study, betamethasone was the most commonly used drug (64.2%)^5^. The steroid use may vary depending on the country, region, and personal preference of the hospital medical staff in which the study was conducted.

*Pseudomonas* spp. were the most common in the PS group. Other studies have reported varying results regarding the frequently isolated microorganisms in steroid-treated groups. In one study of contact lens bacterial keratitis, *P. aeruginosa* was reported to be the most common pathogen in the steroid and non-steroid groups^26^. A Japanese study reported that *S. epidermidis* is the most common bacterium in steroid-exposed eyes^5^. Previous experimental studies have reported that certain strains proliferate better with steroid use^4,20,28,29^. One animal experimental study reported that steroids enhance the stromal growth of some bacteria, such as *Pseudomonas* spp., but not *Staphylococcus* spp. and *Streptococcus* spp^29^.

In this study, previous OSD was significantly more common in the PS group (41.2%) than in the NPS group (23.8%). In addition, the proportion of patients with previous OSD was 70.6% in the long-term group, which was significantly higher than that in the short-term (23.5%) and NPS (23.8%) groups. These findings may be related to the prior long-term use of steroids to treat underlying ocular surface conditions. Other studies have also reported a more frequent history of OSD in the PS group^19,25,27^. In the PS group, herpetic keratitis was the most common, accounting for 33.3% (4/14) of previous OSD. Similarly, Otri et al. reported that herpetic keratitis (42.6%) was the most common OSD in patients with sight-threatening infectious keratitis^27^. In bullous keratopathy, topical steroid use has been reported as a significant risk factor for the development of ulcerative keratitis (odds ratio 2.63, 95% confidence interval = 1.41–4.91)^30^ Notably, corticosteroids increase the risk of infection in eyes with OSD by suppressing innate corneal epithelial defense^30–33^.

Poor initial clinical characteristics, including poor initial BCVA (< 0.1, Snellen) and EDS of ≥ 5 mm^2^, were more common in the PS group than in the NPS group, which is consistent with the similar studies. Wong et al. also reported that the median diameter of epithelial defect in steroid-treated eyes was significantly larger (3.5 mm vs. 2.0 mm, *p* = 0.031)^34^. Wang et al. reported that patients with prior steroid use had a 7.7-fold increased risk of developing larger ulcers (*p* = 0.033)^26^. In general, topical steroid use may reduce the initial inflammatory response in infectious keratitis. However, if antibiotic treatment is not sufficiently administered simultaneously in early keratitis or if topical steroids are stopped after the infection is recognized, it can lead to rapid inflammation and disease progression. These mechanisms are thought to contribute to worse initial clinical characteristics in the PS group. In addition, a higher proportion of patients with previous OSD in the PS group than in the NPS group may have contributed to the higher proportion of patients with poor initial clinical characteristics in the PS group.

In terms of treatment outcomes, EHT > 14 days was more common in the PS group (55.9% vs. 37.3%, *p* = 0.045). Delayed epithelial healing has been similarly reported in previous studies evaluating the effectiveness of a combination of antibiotics and topical steroids in infectious keratitis^9,35,36^. In one prospective study, randomized administration of topical steroids in culture-positive bacterial keratitis significantly delayed re-epithelialization in the steroid-treated group compared with that in the placebo group^9^. Chung et al. reported that topical steroid treatments had a negative effect on keratitis by exacerbating infection due to severe inflammatory side effects and also had an effect on microbial replication and delayed epithelial regeneration^35^. In an experimental study using rabbits, the rate of corneal epithelial regeneration was significantly slower in the steroid-administered group than in the control group^36^. In addition to delayed epithelial healing, the PS group showed relatively poor clinical features and treatment outcomes, which may be related to the high prevalence of previous OSD in the PS group. This is because in the case of previous OSD, poor visual acuity and poor treatment results may be more common due to pre-existing corneal lesions and opacities.

Our study showed that prior topical steroid use (*Z* = 2.46) was a significant risk factor for surgical intervention. This may be related to the relatively high incidence of delayed epithelial healing and impending corneal perforation in the prior topical steroid use group. Many studies have reported an increased risk of subsequent complications in bacterial keratitis associated with previous topical steroid use, including indolent ulceration^17^, perforation^14,18^, endophthalmitis^37^, and treatment failure.^15,20^ Coster et al. reported a higher proportion of successful treatment outcomes in patients without steroid use (78%) than in those who received steroids (69%)^38^. In addition, other significant risk factors for surgical intervention in the total study population were deep infiltration, diabetes, old age, and large EDS. These findings are similar to those of many other studies of bacterial keratitis that reported that the severity of the initial clinical findings and an older age were important risk factors for poor treatment outcomes^27,39^.

The risks of chronic topical corticosteroid use in ophthalmology, such as glaucoma and cataract development, are well known^40^. However, studies on the risk of long-term steroid use compared with short-term use in bacterial keratitis remain lacking. This study found that long-term steroid use was a significant risk factor for surgical intervention in the total group. Further, no significant differences in the treatment results between the long- and short-term groups, except for OSD and epithelial defect size, were observed. Interestingly, the short-term group accounted for half of the PS group. In the short**-**term group, most patients were referred to our hospital because steroids were used after the onset of keratitis symptoms, and the infection worsened afterwards. Therefore, physicians should be cautious in the early use of steroids for keratitis.

To our knowledge, few studies have reported clinical differences in infectious keratitis according to the potency of the topical steroid used. Of total groups, the strong steroid group was one of the significant risk factors for surgical intervention. No significant difference between the strong and weak steroid groups, except for delayed hospital visit and delayed epithelial healing time, was observed. These slight differences may be related to the stronger anti-inflammatory actions of steroids with greater potency. However, there are some limitations in interpreting the results according to the potency of steroids because the daily doses of all the steroids used in this study could not be investigated.

Topical steroids reduce inflammation in many ocular diseases. In the case of infectious keratitis, the use of topical steroids is contraindicated in fungal and acanthamoeba keratitis^5,19,41^. For bacterial keratitis, there have been discussions and literature reports on the therapeutic effect of topical steroids as adjuvants^7,42^. In clinical practice, determining the cause of infection until culture results are obtained is not always possible, and topical steroids can cause and exacerbate infections. Therefore, the perspective of this study, which focused on the disadvantages rather than the advantages of topical steroids, remains important for physicians.

This study had several limitations. First, this was a retrospective study of patients at a single tertiary referral hospital in South Korea. In addition, the difference in the ratio of the PS and NPS groups was not adjusted through a case–control match because the purpose of study was to present all consecutive data during the study period. Second, as this study analyzed only inpatients, it included patients with relatively more severe symptoms and signs. Therefore, the results of this study cannot be generalized to other population groups. Third, a detailed analysis according to various previous OSD etiologies could not be performed because of the smaller number of patients in each group and individual differences. Despite these limitations, this study highlights the risk of corneal infection exacerbation and the side effects of prior topical steroid use in clinical practice. In the future, a multicenter study can be considered to obtain more reliable results using a case–control matching method in larger dataset.

In this study, no significant difference in the distribution of causative isolates between the PS and NPS groups was observed. The PS group had a higher proportion of patients with previous OSD, worse initial clinical characteristics, a longer epithelial healing time, and more surgical treatments than the NPS group. Considering that topical corticosteroid used can be controlled by a physician, close monitoring with frequent regular follow-ups is necessary when administering topical steroid therapy for ocular surface diseases.

## Data Availability

The datasets generated during and/or analyzed during the current study are available from the corresponding author upon reasonable request.
